# Controlled Deuteration of Phosphatidylcholine Biosynthetically Produced from Engineered *Escherichia coli* for SANS Studies

**DOI:** 10.1021/acsomega.6c05455

**Published:** 2026-08-10

**Authors:** Qiu Zhang, Matthew J. Keller, Wellington Leite, Robert L. Hettich, Hugh O’Neill, Hong-Hai Zhang

**Affiliations:** † Neutron Scattering Division, 6146 Oak Ridge National Laboratory, Oak Ridge, Tennessee 37830, United States; ‡ Biosciences Division, Oak Ridge National Laboratory, Oak Ridge, Tennessee 37830, United States; § University of Tennessee, Knoxville, Tennessee 37996, United States; ∥ Center for Nanophase Materials Sciences, Oak Ridge National Laboratory, Oak Ridge, Tennessee 37830, United States

## Abstract

Deuterated phosphatidylcholine (PC) was produced from engineered *Escherichia coli* (*E. coli*) BL21 cells grown in deuterated media. The levels of deuteration could be controlled by growing cells in different combinations of deuterated and nondeuterated water, glycerol, and choline, which were analyzed by NMR, mass spectrometric analysis, and small-angle neutron scattering (SANS). By growing cells in deuterium oxide (D_2_O) in the presence of fully deuterated glycerol and choline, perdeuterated PC (PC-DDD) was produced via this method. The average neutron contrast match points of isolated PC were experimentally determined using SANS. For partially deuterated PC lipids, SANS showed match points of 92 ± 8% D_2_O for lipids with deuterated fatty acyl chains and glycerol (PC-DDH) and 81 ± 2% D_2_O for lipids with only deuterated fatty acyl chains (PC-DHH). Our results will benefit SANS studies that utilize deuterated PC lipids as mimetics of lipid bilayers or membrane-like models for structural studies of biomolecular systems.

## Graphical abstract

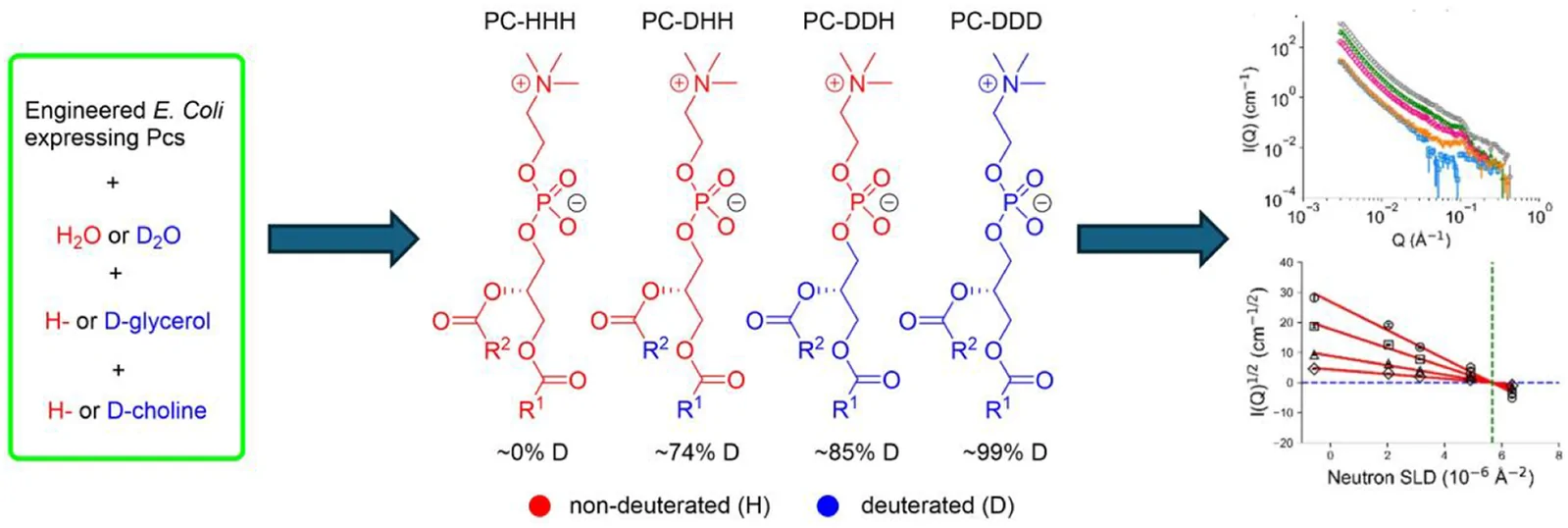

## Introduction

Neutron scattering is a powerful tool in biophysical research because hydrogen and its isotope deuterium have different neutron scattering properties, providing a unique source of experimental contrast that is not available with other structural techniques.^1–3^ Biological macromolecules, including lipids, proteins, and nucleic acids, have distinct neutron scattering length densities (nSLDs) that arise from their differing atomic compositions, particularly their hydrogen content.^4,5^ These differences make it possible to distinguish the structural contributions of individual molecular components within complex biological assemblies. This contrast can be further manipulated by varying the ratio of deuterium oxide (D_2_O) to H_2_O, allowing the nSLD of the solvent to be matched to that of specific components and thereby selectively suppressing or enhancing their scattering contributions.^6–10^ However, solvent contrast variation alone is often insufficient for systems composed of chemically similar molecules with comparable nSLDs. In these cases, selective deuterium labeling provides an additional level of contrast that enables individual components to be resolved. This approach is particularly important for studies of heterogeneous lipid membranes, where different lipid species often possess nearly identical nSLDs and cannot be distinguished without isotopic labeling.^11–13^

Phospholipids are an important target for deuterium labeling in membrane-related studies of many biological processes, including cell recognition, signaling, selective ion transfer, adhesion, and membrane fusion.^14^ Currently, there are three main ways to obtain deuterated phospholipids: biodeuteration, chemical deuteration, or semisynthetic approaches that combine both routes. Chemical deuteration has been established as an efficient method to obtain partially labeled and perdeuterated phospholipids with saturated fatty acyl tails, directly by the condensation of deuterated fatty acids and glycerol phosphate.^15^ However, phospholipids with saturated fatty acyl tails are not representative of the phospholipids found in biological membranes under physiological conditions, since most biological phospholipids are heteroacyls, containing one saturated fatty acyl chain and one unsaturated fatty acyl chain. Although the chemical synthesis of deuterated fatty acyl chains containing one or two unsaturated bonds has been reported, its complicated synthesis renders it impractical for routine preparation,^16,17^ and a semisynthetic approach combining chemical deuteration and biosynthesis has been developed for the preparation of partially deuterated phosphatidylcholine (PC) with one saturated tail and one unsaturated tail; however, this approach still suffers from time-consuming multistep chemical deuteration of unsaturated fatty acids with an overall low yield.^18^ Chemical deuteration and semisynthetic approaches are able to provide uniformly labeled phospholipids for preparing model bilayers. While model systems are useful for investigating fundamental behaviors of cell membranes, research on native cell membranes or native-like lipid mixtures to elucidate real biological systems has recently attracted more attention.^19^ In this context, biological deuteration provides an alternative way to produce deuterated total lipid extracts. The deuterated lipids can be extracted from the organism grown in minimal media with defined levels of D_2_O that can include deuterated carbon sources such as glycerol or glucose. Deuterated phospholipids obtained from this approach have been widely used in the study of interactions between phospholipids and other biomolecules, including sterols, peptides, and proteins.^20–22^

While the biological synthesis of deuterated total lipid extract has been well developed,^23^ preparation of deuterated PC is still challenging due to the lack of PC in natural prokaryotic membranes.^24,25^ On the other hand, PC is a major component of biological membranes in many eukaryotic cells, and it is an essential part of mimicking native-like membranes for biophysical studies. Therefore, many efforts have been made to produce PC, and using genetically modified yeast is one of the most efficient methods. In 2014, Jouhet’s group reported the preparation of deuterated PC in the methylotrophic yeast *Pichia pastoris*.^26^ In the following year, the Pomorski group successfully produced deuterated PC with *Escherichia coli* (*E. coli*) strain AL95.^27^ As the combination of genetic modification of the microorganism and exposure of the cultures to deuterium oxide can change the phospholipid composition in the plasma membranes, it is important to identify the isolated PC for downstream characterization studies. Herein, we report our study on extraction and purification of deuterated PC from *E. coli* that express *Pseudomonas fluorescens* PC synthase (pf-Pcs) and its characterization by MS and SANS analysis.

## Materials and Methods

### Chemicals

Ethyl oxamate, choline, lithium aluminum deuteride (LiAlD_4_), deuterium oxide (D_2_O), methyl-*d*_3_ iodide (CD_3_I), silver oxide (Ag_2_O), sodium hydroxide (NaOH), ethanol (EtOH), and anhydrous tetrahydrofuran (THF) were purchased from Sigma-Aldrich and used as received without any further purification. Total lipid extract was purchased from Avanti Polar Lipids.

### Strains and Culture Conditions

To produce PC in *E. coli*, the pET24b plasmid containing the gene encoding pf-Pcs was transformed into *E. coli* BL21­(DE3). Initially, 5 mL of Luria–Bertani (LB) broth was inoculated from a single colony and incubated at 37 °C overnight. The cell cultures were then adapted to grow in D_2_O Enfors-based minimal media [25] with either H-glycerol or D-glycerol as the carbon source. The cells underwent three exchanges of media to ensure complete adaptation from H_2_O- to D_2_O-based media. For each exchange, 250 μL of cell culture was centrifuged at 4000*g* for 10 min and the cell pellet was resuspended in 5 mL of the appropriate fresh media and incubated at 37 °C until the cell density at 600 nm (OD_600nm_) reached the stationary phase (OD_600nm_ = ∼5). After the final exchange, 1 L of culture was grown in the H or D Enfors minimal media supplemented with H or D glycerol and H or D choline, respectively, with initial inoculation at OD_600nm_ = ∼0.1. The cells were induced with 0.1 mM isopropyl-β-d-thiogalactopyranoside (IPTG) to switch on expression of Pcs at OD_600nm_ = 0.8–1. Protiated or deuterated choline (1 mM final concentration) was added at this time, and cell growth was continued at 30 °C for 18 h. The cells were harvested by centrifugation at 6000*g* for 30 min and then washed with 1% NaCl three times, followed by freeze-drying for 48 h. The final dry weight was ∼1.1 g cells/L culture. The freeze-dried cells were stored at −80 °C.

### Separation of Total Lipid Extract

*E. coli* total lipids were extracted using the modified Bligh and Dyer method.^28^ In brief, 250 mL of chloroform, 500 mL of methanol, and 200 mL of water were sequentially added to the freeze-dried cells (1.1 g) to achieve a final chloroform/methanol/water ratio of 1:2:0.8 (v/v/v). The cells were vortexed for 15 s immediately following the addition of each solvent and allowed to stand for approximately 18 h at room temperature with stirring. This was followed by addition of chloroform (250 mL) and water (250 mL) to obtain a final chloroform/methanol/water ratio of 1:1:0.9 (v/v/v) to separate the aqueous and organic fractions of the mixture. A separatory funnel was used to collect the lower chloroform phase, which contains the total lipid extract. The chloroform–methanol mixture was then evaporated under reduced pressure using a rotovap (Buchi, R-205). The lipid residue was dried under vacuum overnight to obtain 34 mg of yellow solid.

### Fraction of Total Lipid Extract for PC Purification

Total lipids were separated using silica gel chromatography with chloroform:methanol:water (65:25:4) as the mobile phase. The structure and deuteration level of lipids were confirmed by ^1^H NMR. The naming scheme for the position of the deuterium in the PC lipid is represented in the following order: tail – glycerol – headgroup.

Nondeuterated PC grown from H-medium: yield: 11 mg from 1.6 g of dry cell (PC-HHH).

^1^H NMR (500 MHz, CDCl_3_): δ 5.37–5.33 (m, 2 H), 5.22–5.15 (m, 1H), 4.40–4.37 (m, 1 H), 4.31 (s, 2H), 4.13–4.09 (m, 1 H), 3.97–3.88 (m, 2 H), 3.79 (s, 2 H), 3.43 (s, 9 H), 2.31–2.25 (m, 4 H), 2.02– 1.99 (m, 4 H), 1.59–1.55 (m, 4 H), 1.37–1.25 (m, 44 H), 0.89–0.86 (m, 6 H).

Fatty acyl tail-deuterated PC grown from D_2_O: yield: 9 mg from 0.56 g of dry cell (PC-DHH).

^1^H NMR (500 MHz, CDCl_3_): δ 5.33 (s, **labeled, 0.09 H**), 5.21–5.16 (m, 1 H), 4.39–4.37 (m, 1 H), 4.30 (s, 2 H), 4.13–4.09 (m, 1 H), 3.94–3.89 (m, 2 H), 3.79 (s, 2 H), 3.33 (s, 9 H), 2.31–2.26 (m, **labeled, 0.12 H**), 1.95 (s, **labeled, 0.14 H**), 1.53–1.51 (m, **labeled, 0.37 H**), 1.25–1.19 (m, **labeled, 3.69 H**), 0.85–0.82 (m, **labeled, 0.91 H**). Average deuteration calculated from ^1^H NMR: 72%

Fatty acyl tail-glycerol linker-deuterated PC grown from D_2_O and D-glycerol: yield: 6 mg from 0.46 g of dry cell (PC-DDH).

^1^H NMR (500 MHz, CDCl_3_): δ 5.33 (s, **labeled, 0.01 H**), 5.16 (**labeled, 0.00 H**), 4.45 (**labeled, 0.02 H**), 4.31 (s, 2 H), 4.09 (m, **labeled, 0.04 H**), 3.92 (m, **labeled, 0.04 H**), 3.81 (s, 2 H), 3.36 (s, 9 H), 2.25–2.22 (m, **labeled, 0.05 H**), 1.96 (s, **labeled, 0.04 H**), 1.56–1.48 (m, **labeled, 0.08 H**), 1.25–1.19 (m, **labeled, 1.16 H**), 0.89–0.82 (m, **labeled, 0.30 H**). Average deuteration calculated from ^1^H NMR: 82%

Choline head-deuterated PC grown with D-choline from H_2_O and H-glycerol: yield: 11 mg from 0.75 g of dry cell (PC-HHD).

^1^H NMR (500 MHz, CDCl_3_): δ 5.35–5.33 (m, 2 H), 5.20 (s, 1 H), 4.40–4.37 (m, 1 H), 4.31 (**labeled, 0.00 H**), 4.13–4.10 (m, 1 H), 3.97 (s, 2 H), 3.79 (**labeled, 0.00 H**), 3.43 (**labeled, 0.00 H**), 2.31–2.25 (m, 4 H), 2.03–1.99 (m, 4 H), 1.59–1.55 (m, 4 H), 1.29–1.25 (m, 44 H), 0.89–0.86 (m, 6 H). Average deuteration calculated from ^1^H NMR: 12%

Perdeuterated PC grown from D_2_O and D-glycerol with D-choline: yield: 12 mg from 0.75 g of dry cell (PC_DDD).

^1^H NMR showed only tiny peaks and was unable to be integrated, which suggested >99% average deuterium incorporation.

### Chemical Synthesis of D-Choline

Ethyl oxamate (2.34 g, 20 mmol) was dissolved in anhydrous THF (40 mL) at 0 °C. LiAlD_4_ (1.68 g, 40 mmol) was added portionally while the temperature was maintained at 0 °C. Then, the temperature was slowly raised to 70 °C, and the mixture was stirred overnight. Water (2 mL) was added slowly to quench the reaction. The solid was removed by filtration through Celite and washed with a mixture of methanol and dichloromethane (20 mL:60 mL). The HCl solution (6 N) was added to the filtrate to adjust the pH value to 5. The solvent was evaporated to form the crude product for the next step without any purification. Ethanolamine hydrochloride salt-d_4_ (1.01 g, 10 mmol), iodomethane-*d*_3_ (8.76 g, 60 mmol), and sodium hydroxide (2.8 g, 70 mmol) were dissolved in ethanol/water (48/18 mL), and the mixture was stirred overnight. After the reaction was completed, the solvent was evaporated to dryness. Methanol (30 mL) was added to the residue, and the suspension was filtered to obtain a clear solution. Methanol was removed again by evaporation. Then, water (10 mL) and silver oxide (1 g) were added to the residue, and the mixture was stirred overnight. The white solid was removed by filtration, and the solvent was evaporated to dryness. Water (20 mL) was added to the residue, and the insoluble solid was removed by filtration to obtain a clear solution. The HCl solution (6 N) was added to adjust the pH to 4, and the mixture was kept stirring overnight. The solvent was evaporated to obtain choline-*d*_13_ chloride salt with a final yield of 1.3 g.

Ethanolamine hydrochloride-*d*_4_

^1^H NMR (500 MHz, D_2_O): δ 3.72 (s, **labeled, 0.02 H**), 3.05 (s, **labeled, 0.02 H**). Average deuteration calculated from ^1^H NMR: 99%

Choline hydrochloride-*d*_13_:

^1^H NMR (500 MHz, D_2_O): δ 4.01 (s, **labeled, 0.02 H**), 3.46 (s, **labeled, 0.02 H**), 3.14 (s, **labeled, 0.02 H**). Average deuteration calculated from ^1^H NMR: 99%

### Mass Spectrometric Characterization of Lipid Samples

Samples were redissolved in the “8Bu” solvent mixture (69:23:8 H_2_O:isopropyl alcohol (IPA):butanol (BuOH)) for analysis.^29^ Samples were measured in positive and negative ion modes on a Dionex Ultimate-3000 reversed-phase LC platform coupled online to a Thermo Fisher LTQ-Orbitrap Velos Pro mass spectrometer. This method has been adapted from one developed by Dann-Rasche et al. and employed a 60% acetonitrile and 40% water mobile phase A with a 90% isopropanol and 10% acetonitrile mobile phase B, both containing ∼4.2 g/L ammonium acetate with gradient details in the table below (to facilitate dissolution of ammonium acetate, 1% water may be used in mobile phase B).^29^ Reversed-phase columns with an inner diameter of 100 μm were packed in-house using Kinetex 1.7 μm C18 (100 Å) stationary phase (Phenomenex—Torrance, CA) to a length of about 15 cm. The mass spectrometer was operated with one full scan acquired at 30k resolution, followed by 10 data-dependent fragmentation scans in the ion trap with a collision-induced dissociation normalized collision energy of 30.

#### Lipid Identification

Lipid identifications were performed on the unlabeled samples and propagated to the labeled conditions to avoid identification difficulties arising from more complex isotopic states. These identifications were performed through a manual analysis of the LC–MS/MS data, including chromatographic characteristics and fragmentation spectra. Lipids were annotated according to the LIPID MAPS convention at the species level (reporting the summed tail carbon and unsaturation).^30^ Unsaturated odd-chain lipids were identified and were labeled according to their total carbons and apparent unsaturation without considering if they are cyclopropyl fatty acids (FAs), which generate the same mass and fragment ions in CID fragmentation as true odd-chain unsaturated lipids.^30,31^ The various isotopologues of the identified lipids were quantified in the samples by manual peak area integration using El-MAVEN.^32^

#### Analysis of Lipid Deuteration

Lipid deuteration was calculated following the method described in Keller et al., 2024. Briefly, isotopologue distributions were modeled using a binomial function (eq 1) to characterize the deuterium distribution of the lipids. This assumes that the distributions are generated by stochastic sampling from a mixed pool of protium and deuterium

1 $$P\left(x\right)=\frac{n!}{x!\left(n-x\right)!}{\left(1-p_{D}\right)}^{n-x}{p_{D}}^{x}$$

where *n* = total nonsolvent exchangeable hydrogen, *x* = number of deuterium, *p*_D_ = probability for any nonexchangeable hydrogen to be deuterium, and *P*(*x*) is the probability of generating a lipid with *x* deuterium.

This equation was used to generate expected lipid deuterium distributions by calculating *P*(*x*) for all values of *x* between 0 and *n* (inclusive). The probabilities are also adjusted for the natural isotope distributions of carbon, oxygen, and nitrogen, since the fine isotope structures of the lipids were not resolved. This equation is combined with a Python script,^23^ which fits this equation to the data by optimizing the value of *p*_D_. This returns the deuterium probability for that lipid that best fits the observed data. It corresponds to the percent deuteration of the lipid species. In the case of PC lipids with deuterated media and unlabeled choline, the hydrogen in choline was subtracted from the labelable hydrogen so that an accurate fit could be obtained using eq 1. The resulting *p*_D_ was then scaled according to the total number of nonexchangeable hydrogen. Weighted average deuteration values were calculated by weighting individual lipid species’ deuteration states by their relative abundance within the class.

#### Lipid Class Abundance Calculation

For the PC/PE ratios, peak areas for the relevant species (monoisotopic for the unlabeled condition and all reasonable isotopologues around the mode value for labeled conditions) were integrated in El-MAVEN. The peak areas were scaled based on the ionization efficiencies for PE and PC as follows. A serial dilution of the EquiSplash lipid standard mixture (from Avanti Polar Lipids) was analyzed with the same LC–MS method as the samples. The ratios of the peak areas between the PC and PE standards were obtained across the calibration curve and adjusted on the basis of the molar concentrations of the lipids. These correspond to the relative ionization efficiencies of the different lipid species. Since this can vary at different concentrations, a linear regression was performed on the PC/PE ionization ratio vs PE standard intensity. The linear fit was used to scale the PE peak areas based on where the PE intensity was on the linear fit. For peak areas outside those used in the calibration curve, the end point values of the curve were used. The scaled values were summed, and the ratio of the PC/PE summed abundance was calculated. The values generated are approximate estimates of the molar ratios of the two lipid classes.

### SANS and SAXS Data Collection of Lipid Samples

SANS measurements were performed at the Bio-SANS instrument located at the High Flux Isotope Reactor (HFIR) in the Oak Ridge National Laboratory (ORNL). A standard configuration of the dual detector system was set with the Panel Scan feature, with the main detector at 15.5 m and the wide-angle curved detector at 1.13 m, which was rotated to 1.4°. This instrument setting covered the *Q*-range of 0.003 Å^–1^ to 1 Å^–1^, using a neutron wavelength (λ) of 6 Å with a relative wavelength spread (Δλ/λ) of 13.2%. SANS data were corrected for instrument background, detector sensitivity, and instrument geometry using facility-developed data reduction software, drt-SANS.^33^ All SANS measurements were performed using 1 mm path-length cylindrical quartz cuvettes (Hellma, Müllheim, Germany) at room temperature.

For SANS measurements, lyophilized PC lipid extracts were resuspended directly in the appropriate H_2_O/D_2_O solvent mixtures at 1 mg/mL, homogenized by mixing, and subjected to seven freeze–thaw cycles before being loaded into the sample cells.

For SAXS measurements, the PC samples were prepared by using the same procedure as that for the SANS experiments. The measurements were taken on a Rigaku BioSAXS 2000 instrument.

## Results and Discussion

### Characterization of PC in Total Lipid Extract

Initial experiments analyzed the production of PC in *E. coli* cells expressing *P. fluorescens* Pcs (pf-Pcs *E. coli*). Thin-layer chromatography (TLC) showed the presence of PC, which was then confirmed through LC–MS analysis. As shown in Figure 1A, the chromatographic dimension of the total lipid extract from pf-Pcs-*E. coli* in protiated (HT, third spot from the left) or deuterated media (DT, fourth spot from the left) clearly showed one spot for PC (PC, first spot from the left), while no PC spot was observed in the commercial total lipid extract (CT, second spot from the left). This result indicated that *E. coli* cells expressing pf-Pcs are able to produce PC lipids in both protiated and deuterated media. The phospholipid composition of pf-Pcs-*E. coli* that were grown in protiated media was further analyzed by mass spectrometry, and we found the mass peaks of PC in the total lipid extract. For example, PC 18:1_16:1 was identified from its fragmentation spectra. As shown in Figure 1B, the precursor ion at *m*/*z* 758.65 (758.5676 *m*/*z* in the Orbitrap full scan) confirmed the formula C_42_H_80_NO_8_P. The ion at *m*/*z* 575.57 represented the lipid with phosphocholine neutral loss, which indicated that the lipid is PC. One of the lipid tails was identified by fragment ions at *m*/*z* 522.49 and 504.37, which individually represented the lipid with fatty acid 16:1 ketene neutral loss and carboxylic acid neutral loss. With the same method, the other lipid tail was identified as fatty acid 18:1 by fragment ions at *m*/*z* values of 494.43 and 476.34.

**Figure 1:**
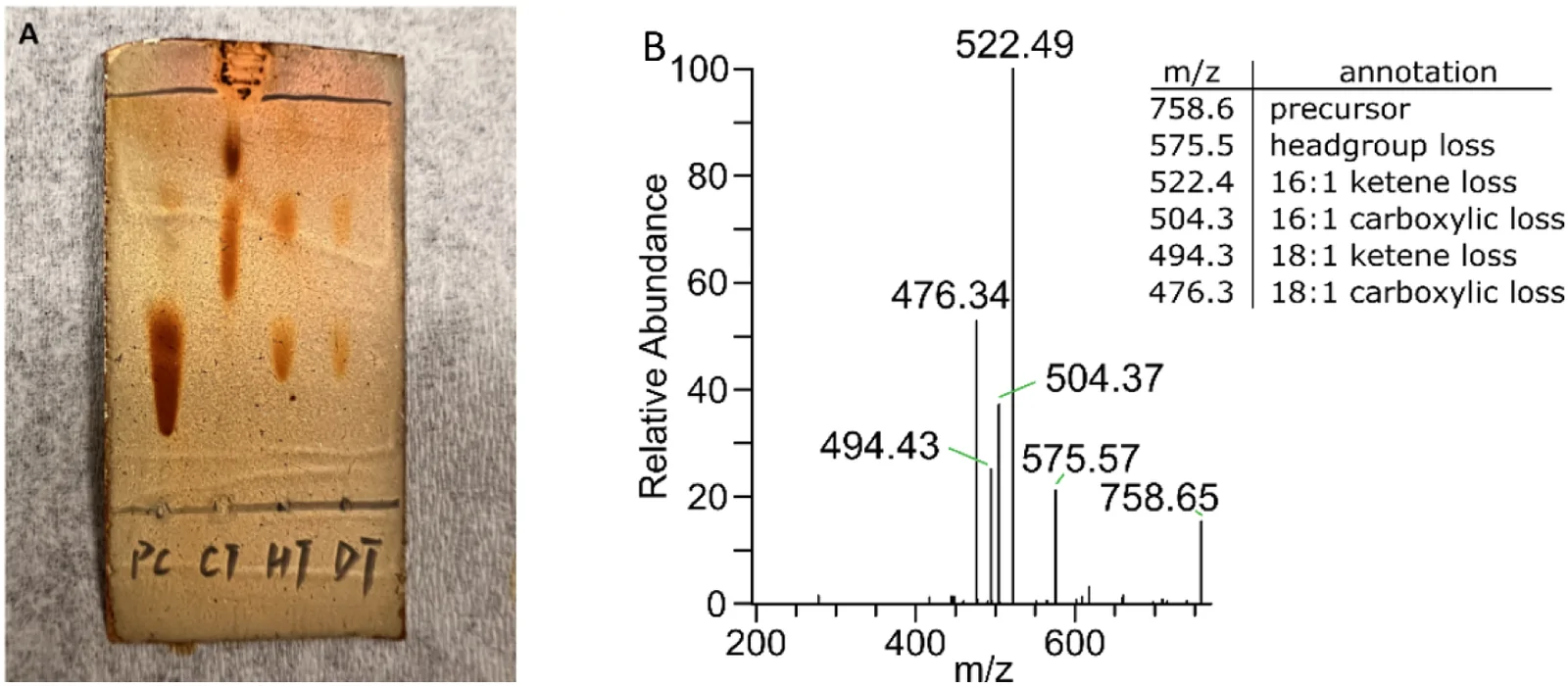
(A). TLC analysis of PC (first spot from left), commercial *E. coli* total lipid extract (CT, second spot from the left), total lipid extract from H_2_O culture (HT, third spot from the left), and total lipid extract from D_2_O-based culture (DT, fourth spot from the left). The total lipid extract was produced and purified following the general method described in Materials and Methods section. (B) Example mass spectrum of identified PC lipids. This lipid is PC 18:1_16:1 identified in the protiated total lipid extract.

### Chain Distribution in PC

Deuterated (or protiated) PC was fractionated and purified using silica gel chromatography with chloroform/methanol/water (65:25:4) as the mobile phase. Five PCs including nondeuterated PC (PC-HHH), fatty acyl tail-deuterated PC (PC-DHH), fatty acyl tail-glycerol linker-deuterated PC (PC-DDH), choline head-deuterated PC (PC-HHD), and fully deuterated PC (PC-DDD) have been isolated via this method. Since the isolated PC is a mixture of lipids with different fatty acid chains and will be used as a model for mimic membranes, it is important to identify its composition in detail. The structure of the isolated PC was first confirmed by ^1^H NMR. The peak at 5.36 ppm represents the hydrogen in the double bond in the fatty acid chain, and the peak at 5.22 ppm represents the hydrogen on carbon 2 of the glycerol backbone. The ratio of integration of these two peaks is 1.80, suggesting that isolated PC contains an average of 0.9 double bonds per PC molecule. To obtain more details of the PC composition, mass spectrometry was employed to analyze the fatty acid chain distributions. While some PC lipid species were not identified under each condition, a total of 21 PC species were identified under all conditions from the LC–MS data. The relative abundances of these species are shown in Figure 2, which indicates that the deuterated growth medium, deuterated glycerol, and deuterated choline can have an impact on the distribution of fatty acid chains. PC samples from deuterium media contain more PC 30:0, PC 32:0, and PC 28:0, which results in the average amount of unsaturated bonds in these PC samples being slightly lower than PC samples from the protiated condition. Although the ratio of some species showed changes, the most abundant species, PC 32:1, PC 34:1, PC 32:2, PC 34:2, and PC 30:1 (which can all be formed from combinations of relatively common fatty acids in *E. coli*), were found in all PC samples. These results suggest that the elongase enzymes may show varied activity in protiated and deuterated environments, leading to changes in PC tail chain distribution.

**Figure 2:**
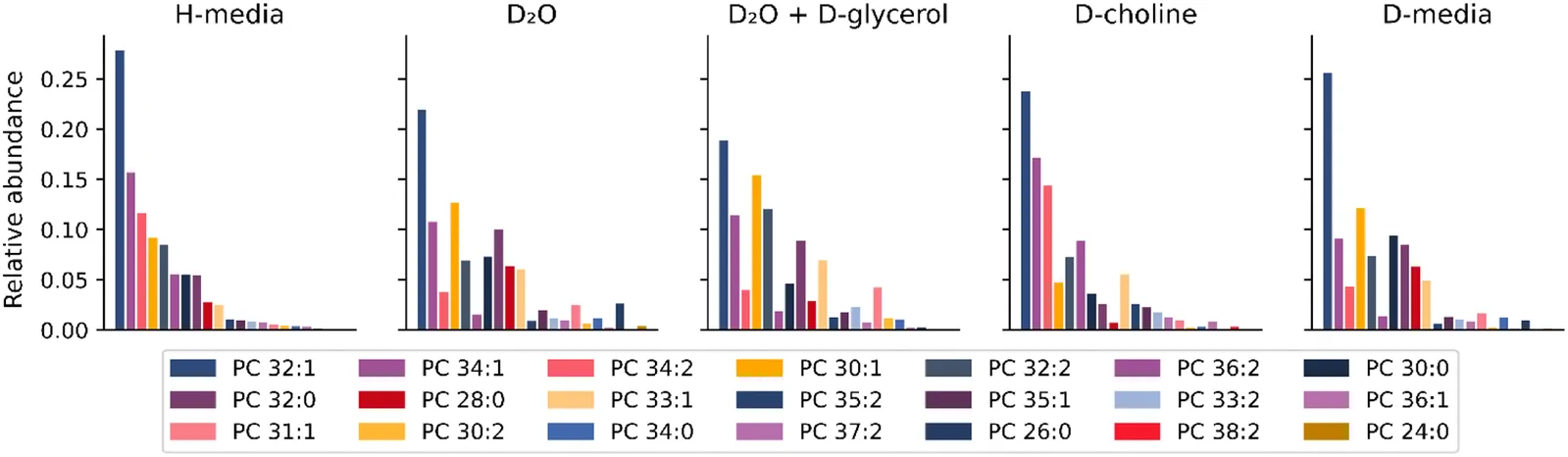
Relative abundance of PC lipids identified in all culture conditions. Values correspond to normalized peak areas associated with each lipid species. Each value was divided by the sum of the individual intensities of each shared lipid.

### Control of Deuterium Label Incorporation in PC

*E. coli* cells grown in D_2_O containing H-glycerol and H-choline produce partially deuterated phosphatidylcholine. NMR analysis shows a significant decrease of proton signal on the fatty acyl chain, indicating deuterium incorporation. The most obvious change is the decrease of the large signal at 1.2 ppm, corresponding to the deuteration of most of the hydrogens on fatty acyl chains. The glycerol signals at 4.0, 4.2, and 5.2 ppm, as well as the signals at 3.4, 3.9, and 4.4 ppm belonging to the choline headgroup, remained the same as the signal of the unlabeled PC molecule. Mass spectrometry of PC extracted from these cells showed that there was an average of 22 residual protium in these lipid species, which is consistent with direct incorporation of H-glycerol and choline into the lipid (for a total of 18 protium) with some glycerol-active protium on hydroxyl groups being incorporated into the fatty acyl tails. Similar labeling trends have been observed for phospholipids in *E. coli* BL21­(DE3) previously.^23^ To investigate the carbon source effect on deuteration, *E. coli* cells were grown in D_2_O-based media containing D-glycerol and H-choline. PC purified from these cells showed only choline group peaks at 3.4, 3.9, and 4.4 ppm, identical to that of unlabeled PC. However, the proton peaks of the glycerol linker at 4.0, 4.2, and 5.2 ppm disappeared in this ^1^H NMR spectrum. These PC species showed an average of 13 protium in the MS data, consistent with full deuteration of the fatty acids and glycerol linker and unlabeled choline. These results show that the proton/deuterium incorporation on PC’s acyl chain is based on the media composition, while glycerol and choline were directly incorporated into PC molecules without modifications. Thus, selective deuteration of PC can be controlled by employing a combination of protiated/deuterated water, glycerol, and choline (Figure 3).

**Figure 3:**
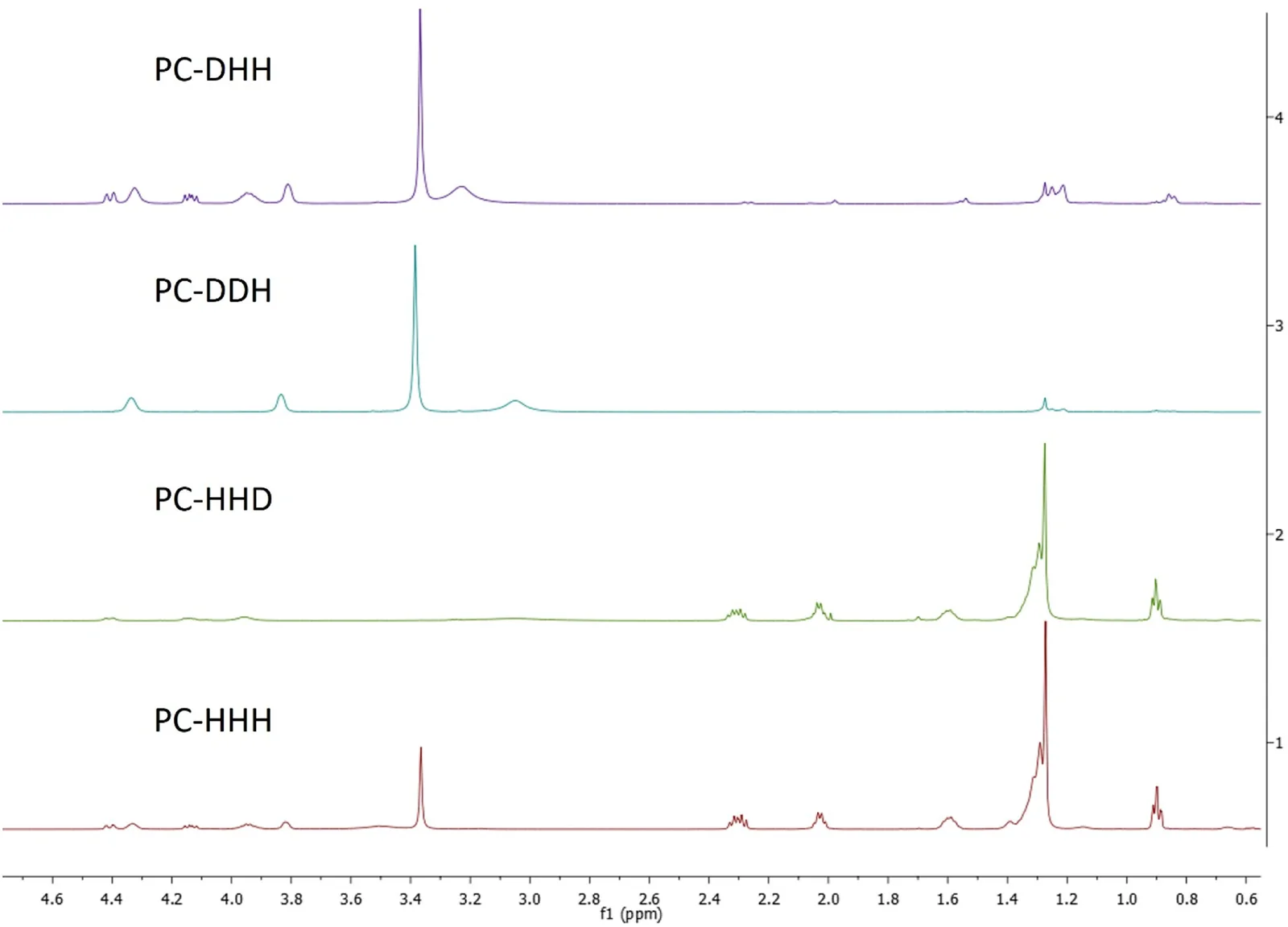
^1^H NMR spectra of isolated PC with different deuteration levels.

### Perdeuterated Choline and PC

Although biosynthesis of deuterated total lipid extract and PC has been explored for many years, perdeuterated PC is still challenging to prepare due to the prohibitive expense of commercially available perdeuterated choline. Therefore, we decided to synthesize perdeuterated choline through chemical deuteration. We adapted a modified method from Yepuri et al.^17^ to obtain a pure product. The deuterium incorporation level of choline was identified as 99% by means of NMR spectra. To test if the perdeuterated choline synthesized in this study behaves the same as the commercial nondeuterated choline, *E. coli* cells were grown in protiated media containing H-glycerol and D-choline. NMR analysis of this PC shows the absence of proton signals from choline at 3.4, 3.9, and 4.4 ppm and the presence of all other proton signals from glycerol and tails. In this context, we prepared fully deuterated PC from *E. coli* cells grown in D_2_O containing D-glycerol and D-choline. NMR analysis of this fully deuterated PC shows only minor peaks (unable to be integrated) at the same positions as those of the unlabeled PC, which indicated its high level of deuterium incorporation. The mass spectrometry analysis of this sample is consistent with the NMR analysis, which showed the deuteration level of fully deuterated PC as 99.6%.

This biological synthetic method has several significant advantages over previous methods for producing deuterated PC. First, this method can produce PC with a controlled level of deuteration ranging from 12% to 99%, by growing cells in different combinations of deuterated and nondeuterated water, glycerol, and choline. For example, PC-DDD was produced by growing cells in deuterium oxide in the presence of fully deuterated glycerol and choline. Therefore, it is a versatile way to selectively target the incorporation of a deuterium label into lipids, offering tailored lipid variants suitable for diverse applications, ranging from fully deuterated to partially deuterated forms. Second, this method can produce PC with mixed tail lengths as a mimic of native cell membranes to elucidate real biological systems. Combining high-resolution MS with NMR analysis enables the characterization of different lipid class distributions and the distribution within individual classes, facilitating the creation of accurate membrane models that better reflect natural systems. Third, this method addresses some of the disadvantages associated with chemical synthetic production methods because it allows for the incorporation of unsaturated acyl chains.^16,17^

### Small-Angle Neutron Scattering Analysis

We used contrast-variation SANS (CV-SANS) to determine the contrast match points of PC lipids and benchmark them against mass spectrometry (MS)–based predictions. MS-derived acyl-chain compositions were used to compute average neutron scattering length densities (nSLDs) and corresponding H_2_O/D_2_O match points. PC-DDD is predicted to be above the solvent-accessible matching range (7.17 Å^–2^ vs ∼6.36 Å^–2^ at 100% D_2_O), whereas partially deuterated PCs match at 92.4% D_2_O (PC-DDH) and 81.9% D_2_O (PC-DHH). PC-HHH shows the lowest predicted match point (14% D_2_O).

CV-SANS measurements yield match points in close agreement with MS predictions. PC-DDD exhibits an average nSLD of 7.2 ± 0.2 Å^–2^, while PC-DDH and PC-DHH match at 92 ± 8% and 81 ± 2% D_2_O, respectively (Table 1 and Figure 4). PC-HHH matches at 22 ± 1% D_2_O, slightly higher than predicted, likely reflecting hydration effects and solvent penetration into the bilayer. Predicted and measured nSLDs show excellent linear agreement (Figure S9). This indicates that MS can be used to reliably estimate nSLDs of deuterated lipids. In addition, the nSLD increases nonlinearly with deuterium incorporation, following an empirical quadratic dependence (Figure S10) consistent with trends reported for other biomacromolecules.^34^ This allows calculation of lipid contrast match points directly from isotopic composition without additional SANS measurements.

**Table 1:** Comparison of Predicted nSLD of Protiated and Deuterated Phospholipid from Mass Spectroscopy (MS) and SANS^a^

		nSLD (Å^–2^)		equivalent H_2_O/D_2_O ratio	
phospholipid extract	deuteration level (%)	MS	SANS	MS	SANS
PC-HHH	0	0.3	0.9 ± 0.2	14.0	22 ± 1
PC-DHH	74	5.1	5.1 ± 0.1	81.9	81 ± 2
PC-DDH	85	5.9	5.7 ± 0.5	92.4	92 ± 8
PC-DDD	100	7.2	7.2 ± 0.2	–	–

a nSLD were also compared in terms its corresponding equivalent in an H_2_O/D_2_O solvent mixture.

**Figure 4:**
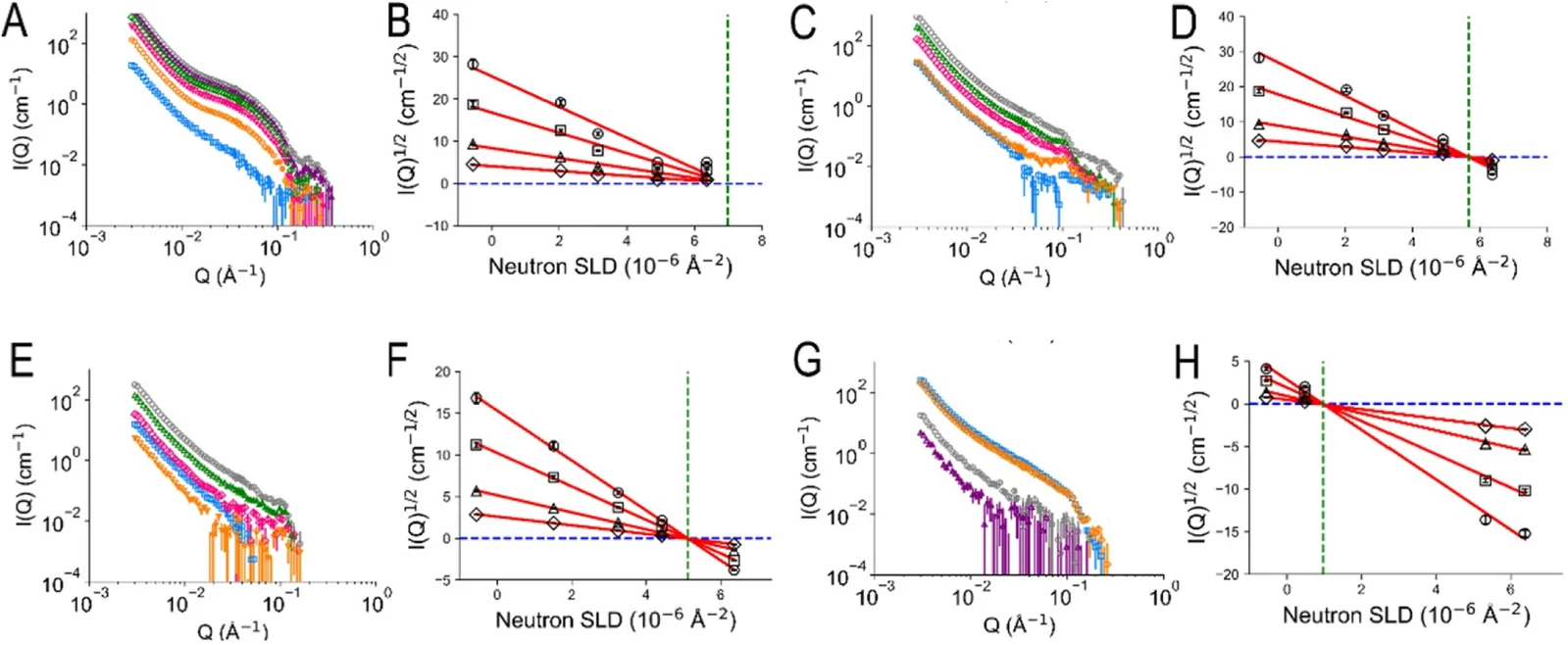
SANS data and contrast match point analysis for deuterium-labeled PC lipids. SANS profiles and contrast match point determination for PC-DDD (A, B), PC-DDH (C, D), PC-DHH (E, F), and PC-HHH (G, H). SANS profiles were measured in 100% D_2_O (blue), 80 or 85% D_2_O (orange), 55 or 50% D_2_O (pink), 40 or 30% D_2_O (green), 20 or 15% D_2_O purple, and 0% D_2_O (gray). The black circles, squares, triangles-up, and diamonds correspond to the square root of the scattering intensity at *Q* points 0.0032 Å^–1^, 0.0039 Å^–1^, 0.0056 Å^–1^, and 0.0085 Å^–1^, respectively. The intersection of dashed blue and red lines indicates the neutron contrast match point for the non- and isotopically labeled phospholipid forms.

At their match points, PC-DDH and PC-DHH exhibit strong suppression of bilayer scattering above *Q* > 0.04 Å^–1^, effectively masking the membrane signal (Figure 5). A weak low-*Q* upturn with a power-law decay of *P* ≈ −3 is consistent with large, compact lipid assemblies or mass-fractal–like heterogeneity, rather than residual bilayer contrast. PC-DDD cannot be contrast-matched using H_2_O/D_2_O alone; however, in principle, mixing deuterated and protiated lipids provides a straightforward route to achieve contrast matching over a broad solvent range (22–100% D_2_O). These contrast conditions enable selective suppression of lipid scattering in nanodiscs and related assemblies and can be used to highlight the scattering signal of membrane proteins and other macromolecules inserted into the bilayer.

**Figure 5:**
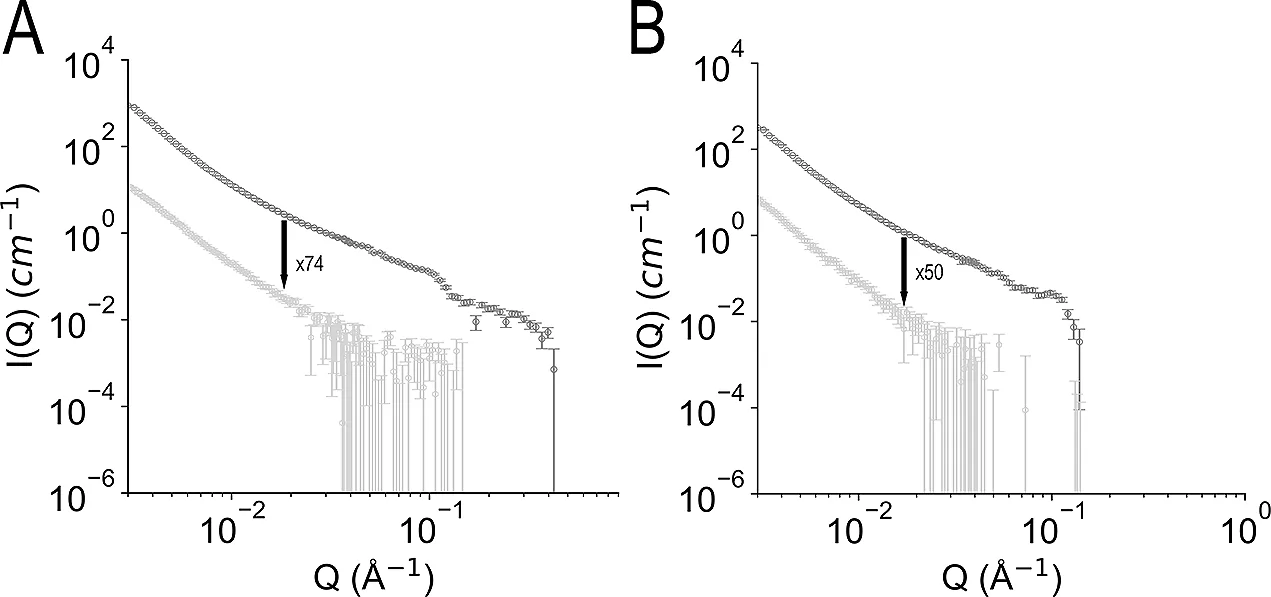
Contrast match points of tail-deuterated and tail-plus-linker-deuterated lipids. (A) PC-DHH; (B) PC-DDH. Dark gray indicates partially deuterated lipids under full contrast conditions (0% D_2_O), while light gray indicates lipids at their average contrast match points in 81% D_2_O and 91% D_2_O buffers, respectively. At contrast match conditions, the bilayer structure disappears, and the remaining scattering arises from large aggregates with inhomogeneous scattering length distributions.

To further probe the bilayer structure of our four PC variants, we fit the SANS data by using a lamellar bilayer form factor implemented in SasView. The form factor describes the internal membrane profile, consisting of a central hydrophobic tail region flanked by hydrophilic headgroup layers, each characterized by distinct thicknesses and scattering length densities. Complementary SAXS measurements of the extracted PC lipid samples indicate multilamellar assemblies for both H- and D-PC lipids, with multiple concentric layers (Figure S11).^35^ Interbilayer correlations arising from this lamellar stacking were captured using a lamellar structure factor, as described elsewhere.^36^

Complementary SAXS measurements of the extracted PC lipid samples indicate lamellar organization for both protiated and deuterated PC lipids, with features consistent with multilamellar assemblies (Figure S11). Importantly, because the samples were freeze-thawed but not extruded or sonicated, the scattering data likely represent heterogeneous hydrated lipid assemblies. Therefore, the SANS/SAXS results should be interpreted as describing the average lamellar organization present in the unextruded hydrated lipid assemblies rather than a unique vesicle morphology.

Across all lipid extracts, the bilayers exhibited similar structural dimensions, with hydrophobic tail thicknesses of ∼10–12 Å and headgroup thicknesses of ∼5 Å. The water volume fraction in the hydrophobic tail region was fixed at 0%, consistent with the highly hydrophobic nature of the bilayer core, while the headgroup region was modeled with partial hydration (50%) (Figure 6).^37^ PC-DDD exhibited a slightly larger lamellar repeat spacing (∼60 Å) compared to the other lipid forms (∼55 Å) (Table 2). For PC-DHH, the data were best described by mixed populations of two- and three-layer stacks (39% *N* = 2/61% *N* = 3). In addition, the SAXS profile of PC-DDD shows a broad lamellar correlation peak at *Q* ≈ 0.10 Å^–1^, corresponding to a repeat distance of ∼61 Å (repeat distance *d* = 2π/*Q*). This agrees well with the SANS-derived lamellar spacing of 59.2 ± 0.9 Å, supporting a multilamellar organization.

**Figure 6:**
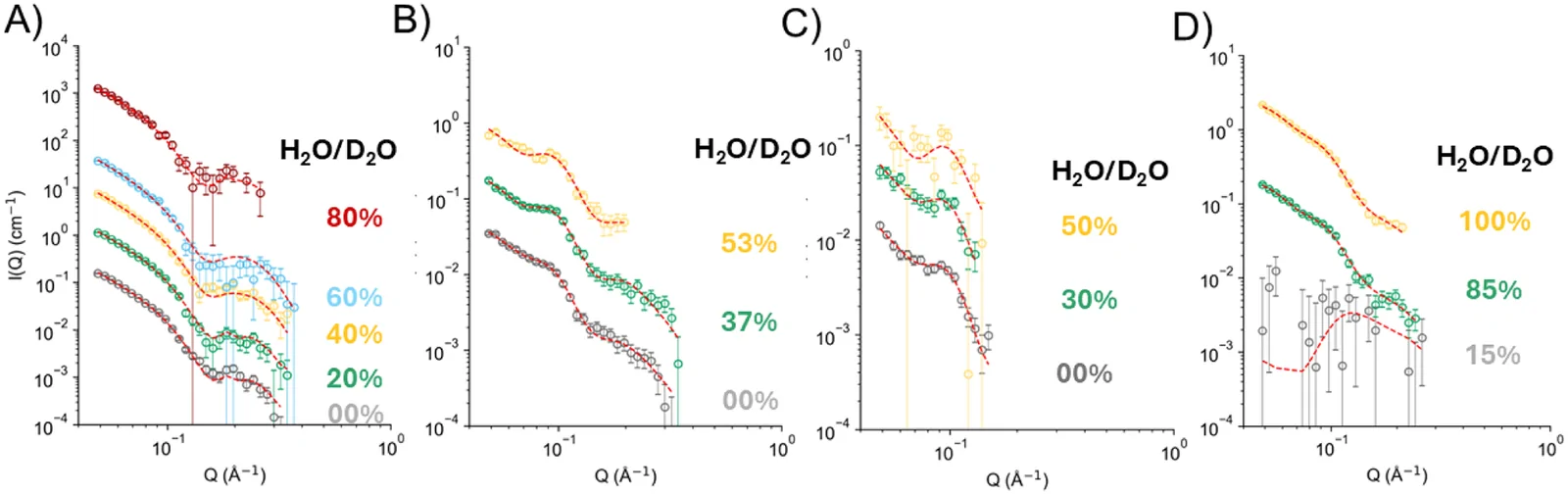
Deuterated PC lipids share common bilayer structural properties. SANS profiles of PC lipid extracts measured at multiple H_2_O/D_2_O solvent contrasts fitted using a lamellar bilayer form factor combined with a lamellar structure factor (red dashed lines). Panels show (A) PC-DDD, (B) PC-DDH, (C) PC-DHH, and (D) PC-HHH. For each panel, the solvent contrasts are color-coded as follows: (A) 80% D_2_O (red), 60% D_2_O (blue), 40% D_2_O (yellow), 20% D_2_O (green), and 0% D_2_O (gray); (B) 53% D_2_O (yellow), 37% D_2_O (green), and 0% D_2_O (gray); (C) 50% D_2_O (yellow), 30% D_2_O (green), and 0% D_2_O (gray);(D) 100% D_2_O (yellow), 85% D_2_O (green), and 15% D_2_O (gray).

**Table 2:** Comparison of Structural Dimensions of Protiated and Deuterated PC

	PC-HHH	PC-DHH	PC-DDH	PC-DDD
deuteration level (%)	0	75	85	100
Hydrophobic Tail				
thickness (Å)	10±8	10±7	12 ± 1	12 ±1
SLD (Å^–2^)^a^	–0.226	5.01	5.73	6.82
% water	0			
Hydrophilic Headgroup				
thickness (Å)^b^	4	4	3.4±0.2	4±1
SLD (Å^–2^)^a^	2.02	2.02	2.38	3.84
% water	50			
Multilayer Proprieties				
*d*-spacing (Å)	54.1±0.6	56.2±1.4	56.4 ± 0.6	59.2 ± 0.9
η	0.8		0.34 ± 0.08	0.6 ± 0.3

a SLD values were constrained on the basis of the deuteration level from MS.

b Head group thickness for protiated and tail-deuterated groups was constrained to similar PC lipids.^38^

The similarity in the average lamellar dimensions is consistent with the lipid composition measured by MS. Although the relative abundance of individual PC species changes across deuteration conditions, the dominant components remain centered around similar PC 30–34 species. Thus, the samples are compositionally similar in their major lipid families but not identical. These mixtures are therefore expected to exhibit broadly similar average lamellar structures, while modest differences in saturation, packing, hydration, and interlamellar interactions may contribute to the small changes observed in the fitted *d*-spacing values.

The structural parameters obtained here can also be compared with literature values for chemically defined synthetic PC bilayers. Synthetic PCs show a clear dependence of hydrocarbon core thickness on acyl-chain length. Short-chain fluid-phase PCs such as DLPC and DMPC have hydrocarbon core half-thicknesses of approximately 10–13 Å, whereas longer-chain PCs such as DPPC, POPC, and DSPC generally show larger values of approximately 14–16 Å. In comparison, the fitted hydrophobic tail half-thicknesses for the biosynthetically produced PC samples are approximately 10–12 Å, placing them closer to the thinner end of the synthetic PC range. This thinner average tail region likely reflects the combined effects of chain-length distribution, unsaturation, and the heterogeneity of the unextruded multilamellar lipid assemblies.

## Conclusion

Understanding the biophysical properties of lipids is crucial for investigating topics involving fundamental studies of bilayer structure and organization, membrane protein structure and function, and advancements in drug delivery and vaccine development.^39,40^ While bacterial lipid production holds distinct advantages over eukaryotic systems in terms of scalability and feasibility for deuteration, differences in lipid classes and their distribution present challenges.^40^ Building upon previous research demonstrating the feasibility of PC production in *E. coli*,^25^ we used this platform to engineer targeted deuterated versions of PC in *E. coli*. In this work, we describe a new method to produce deuterated PC from *Escherichia coli* BL21 cells grown in deuterated media and characterize it with a combination of NMR, mass spectrometric analysis, and small-angle X-ray/neutron scattering.

The technical significance of these findings lies in their ability to fine-tune neutron scattering experiments for complex biological systems. The defined match points for partially deuterated lipids allow for selective minimization of lipid scattering while maximizing the signal from other components, such as membrane proteins. This deuteration strategy is especially advantageous in studying systems such as lipid nanodiscs, where the lipid matrix can be selectively masked to focus on membrane-associated proteins. Additionally, the combination of perdeuterated and protiated PC lipids facilitates SANS experiments that require precise contrast adjustment, making it possible to study membrane dynamics and interactions under varying solvent conditions.

Overall, our study provides a robust approach for the production and characterization of an important isotopically labeled PC lipid that will advance biophysical understanding and pave the way for sophisticated biomembrane-related characterization studies.

## Supplementary Material



## Data Availability

All data will be available to the reviewer or editor upon request.

## Notes

Experimental spectral data (PDF)
