# Supplementary material for: Controlled Deuteration of Phosphatidylcholine Biosynthetically Produced from Engineered Escherichia coli for SANS Studies
Source: ACS Omega. 2026 Aug 10;11(33):50102–11. doi: 10.1021/acsomega.6c05455 (PMC13519996; doi:10.1021/acsomega.6c05455)
Supplement: Supplementary file 1 [file ao6c05455_si_001.pdf]

Controlled deuteration of phosphatidylcholine biosynthetically produced from engineered *Escherichia coli* for SANS study

Qiu Zhang[1], Matthew J. Keller[2,3], Wellington Leite[1], Robert L. Hettich[2], Hugh O'Neill[1*], and Hong-Hai Zhang[4*]

1. Neutron Scattering Division, Oak Ridge National Laboratory, Tennessee, 37830, USA.
2. Biosciences Division, Oak Ridge National Laboratory, Tennessee, 37830, USA.
3. University of Tennessee, Knoxville, Tennessee, 37996, USA.
4. Center for Nanophase Materials Sciences, Oak Ridge National Laboratory, Tennessee, 37830, USA.

Supporting Information

General: NMR spectra were recorded at 23 ºC on a Varian VNMRS 300 MHz NMR spectrometer in $CDCl_3$ unless otherwise noted. Chemical shifts were determined relative to residual $CHCl_3$ (7.26 ppm) for proton, and to the $CDCl_3$ "triplet" at 77.23 ppm for carbon. The D1 relaxation delay is 5 seconds in NMR experiments. The atom% deuterium incorporation was determined by ^{1}H NMR spectrum and MS analysis.

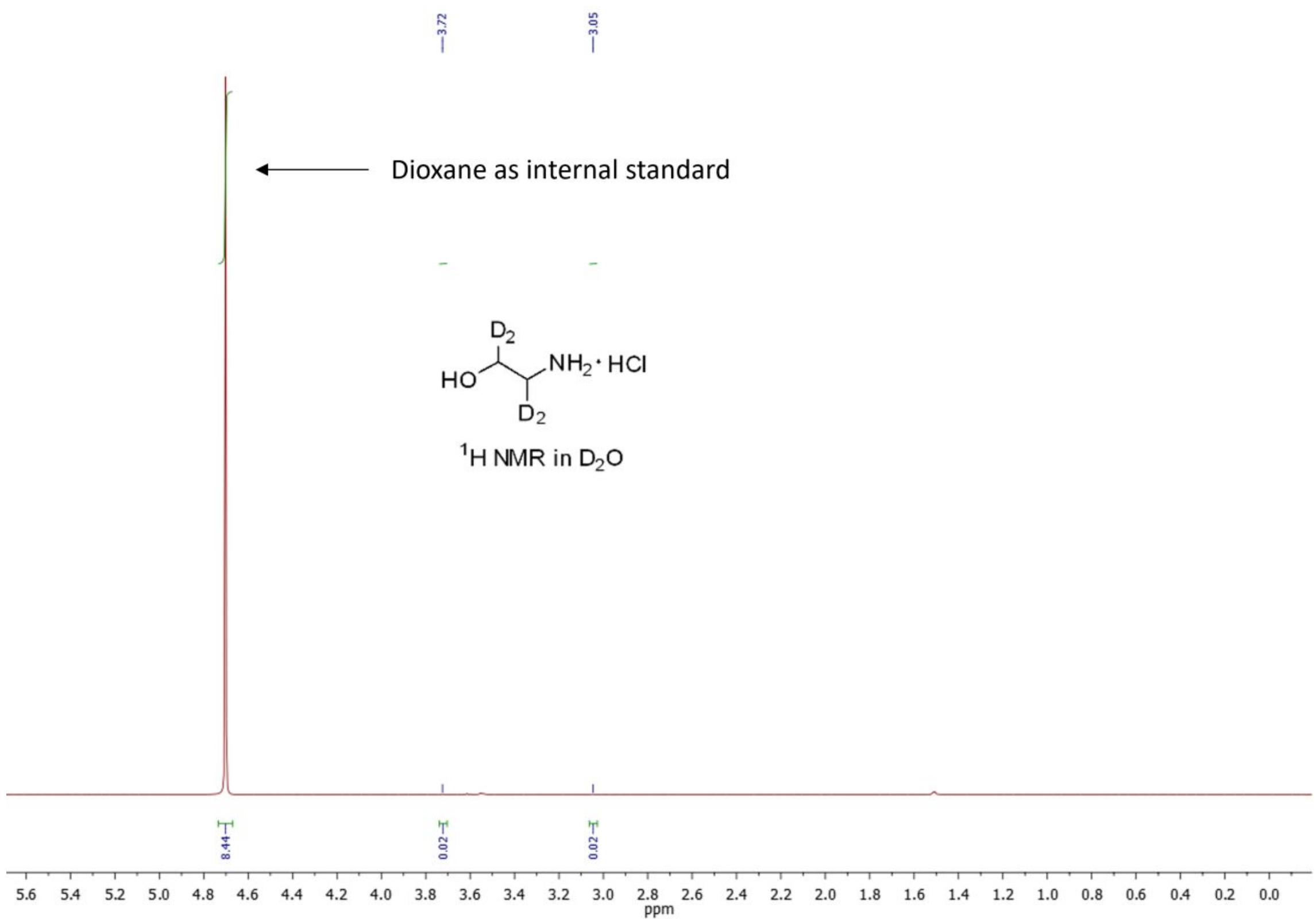

Figure S1. 1H NMR spectrum of aminoethanol-d_4 in D_2O

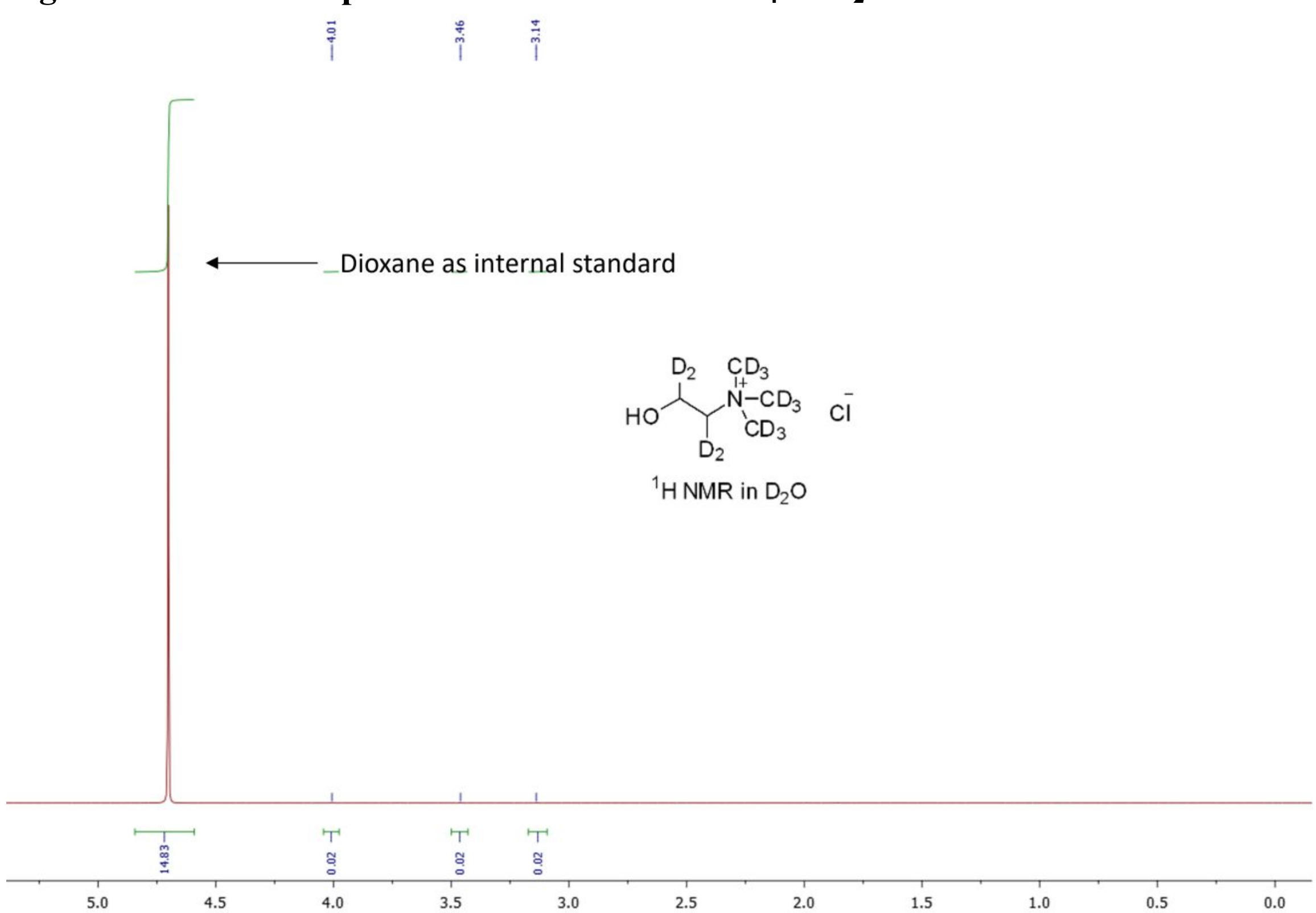

Figure S2. 1H NMR spectrum of choline-d_{13} in D_2O

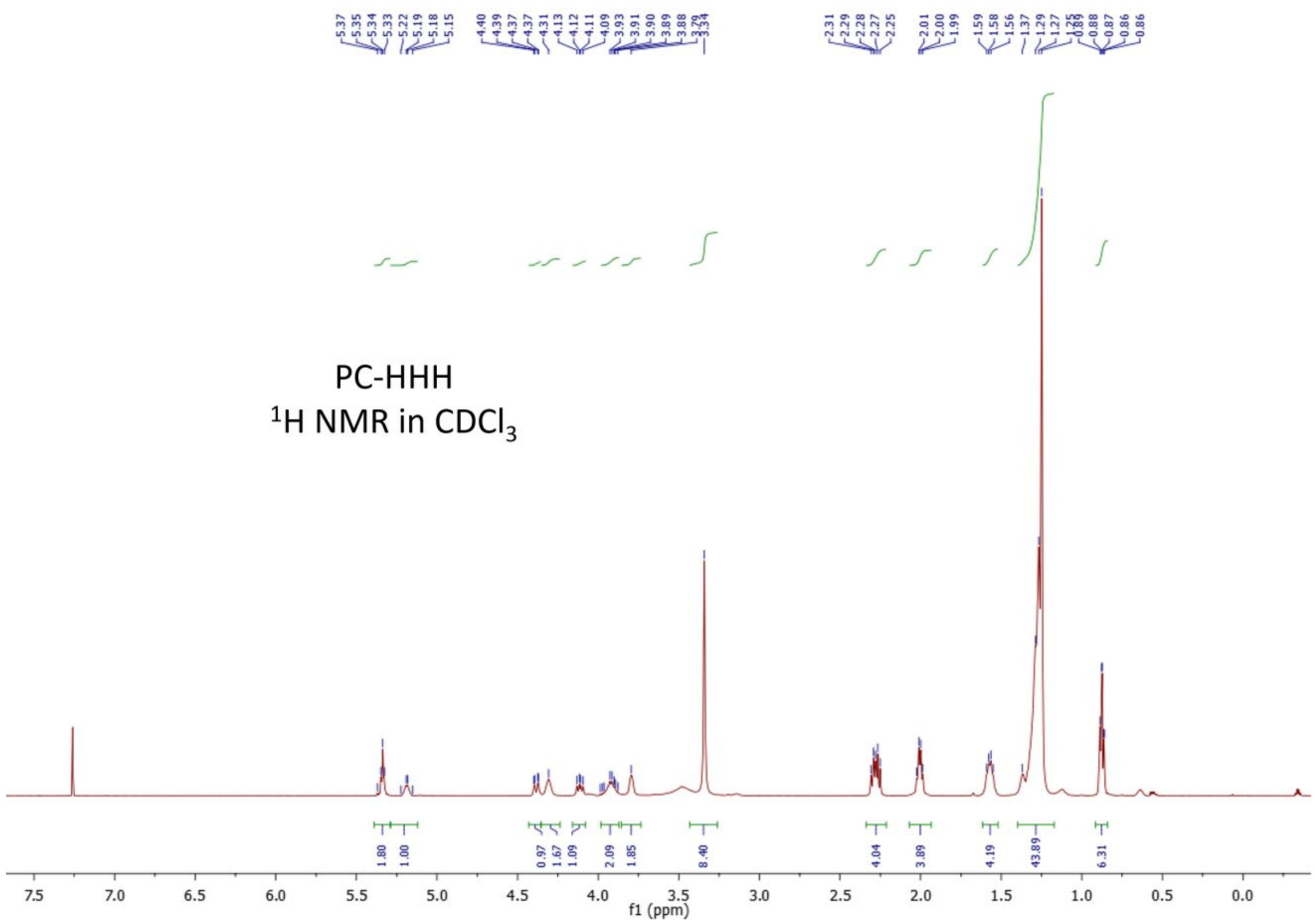

Figure S3. 1H NMR spectrum of PC-HHH in $CDCl_3$

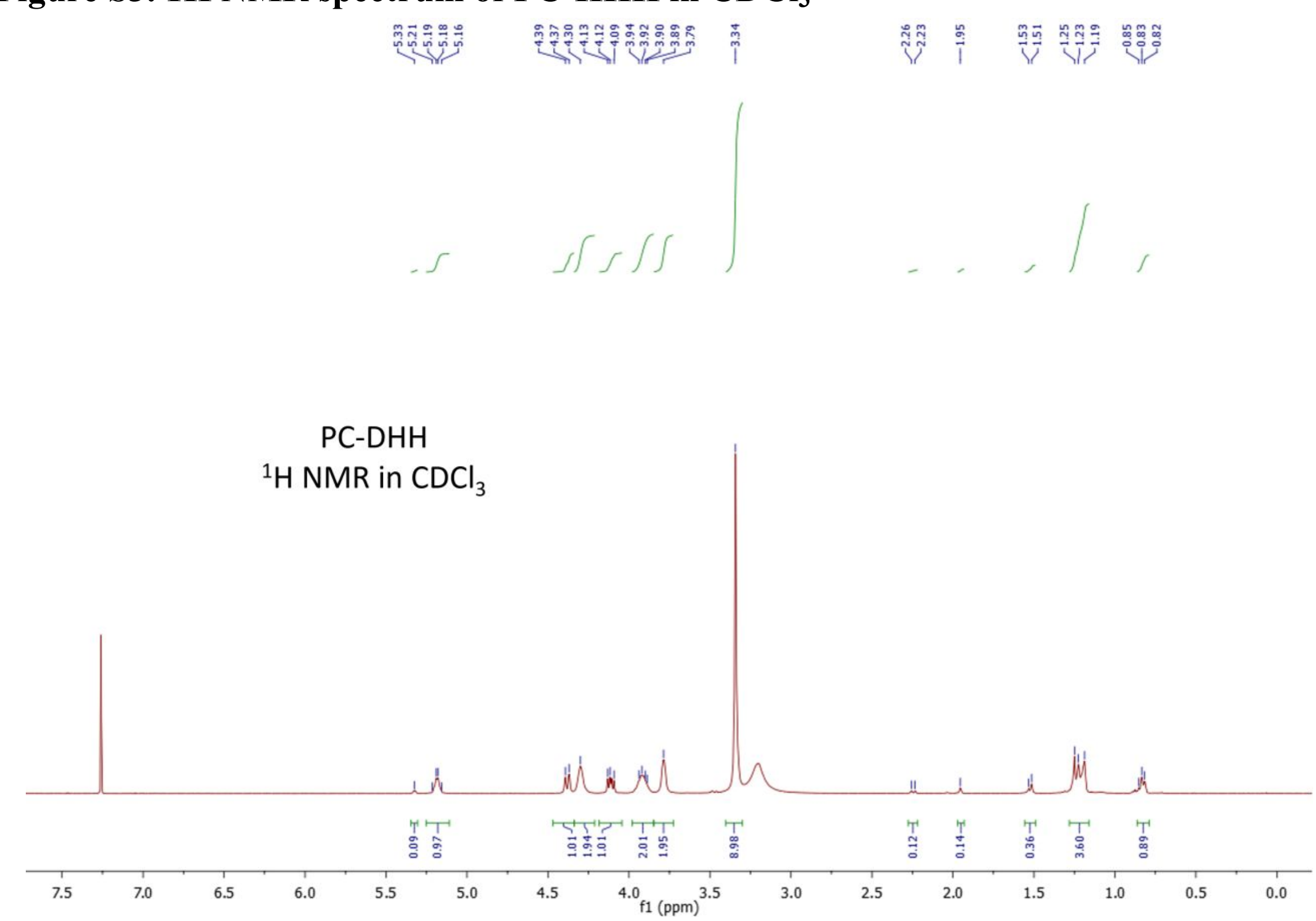

Figure S4. 1H NMR spectrum of PC-DHH in $CDCl_3$

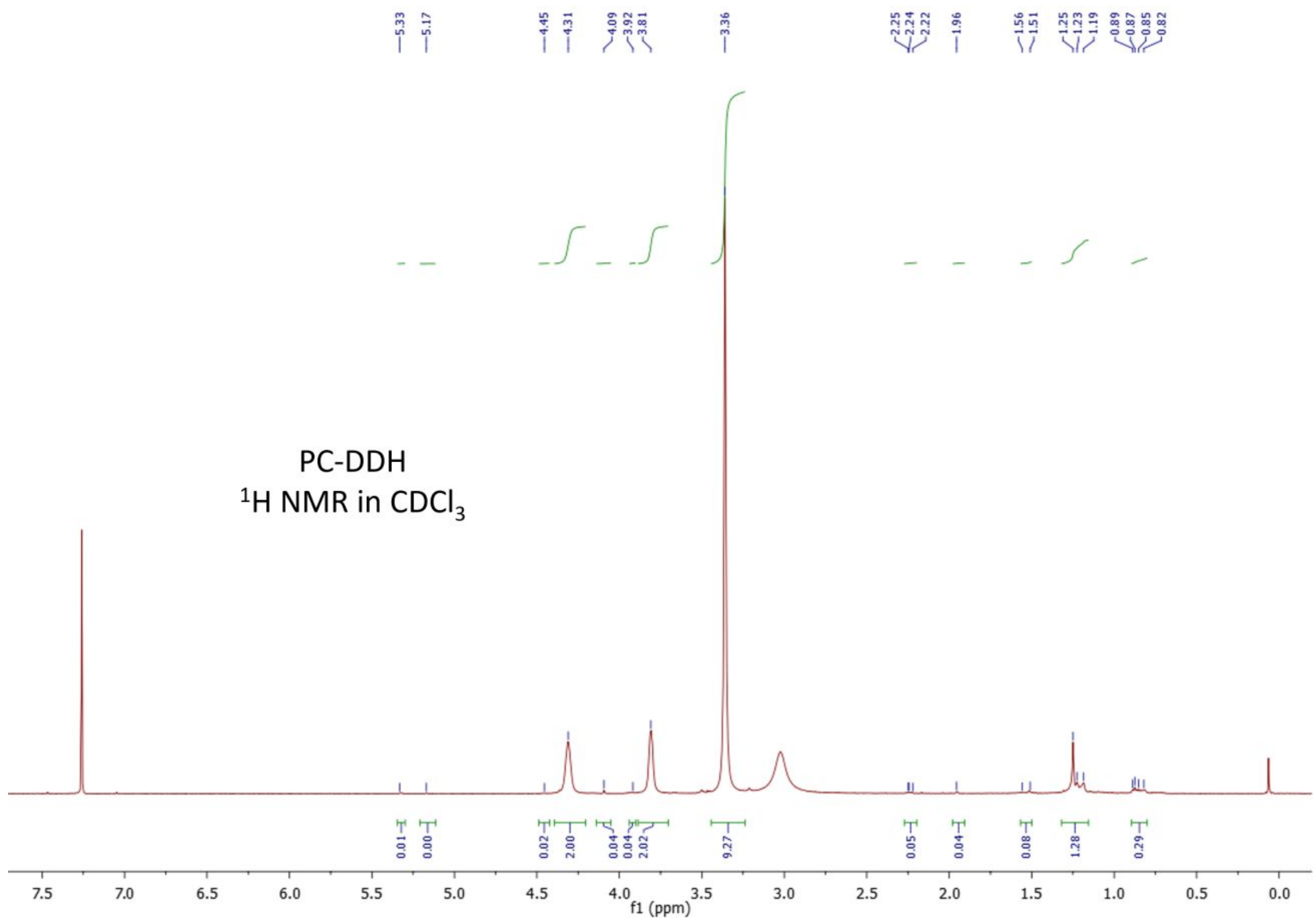

Figure S5. 1H NMR spectrum of PC-DDH in $CDCl_3$

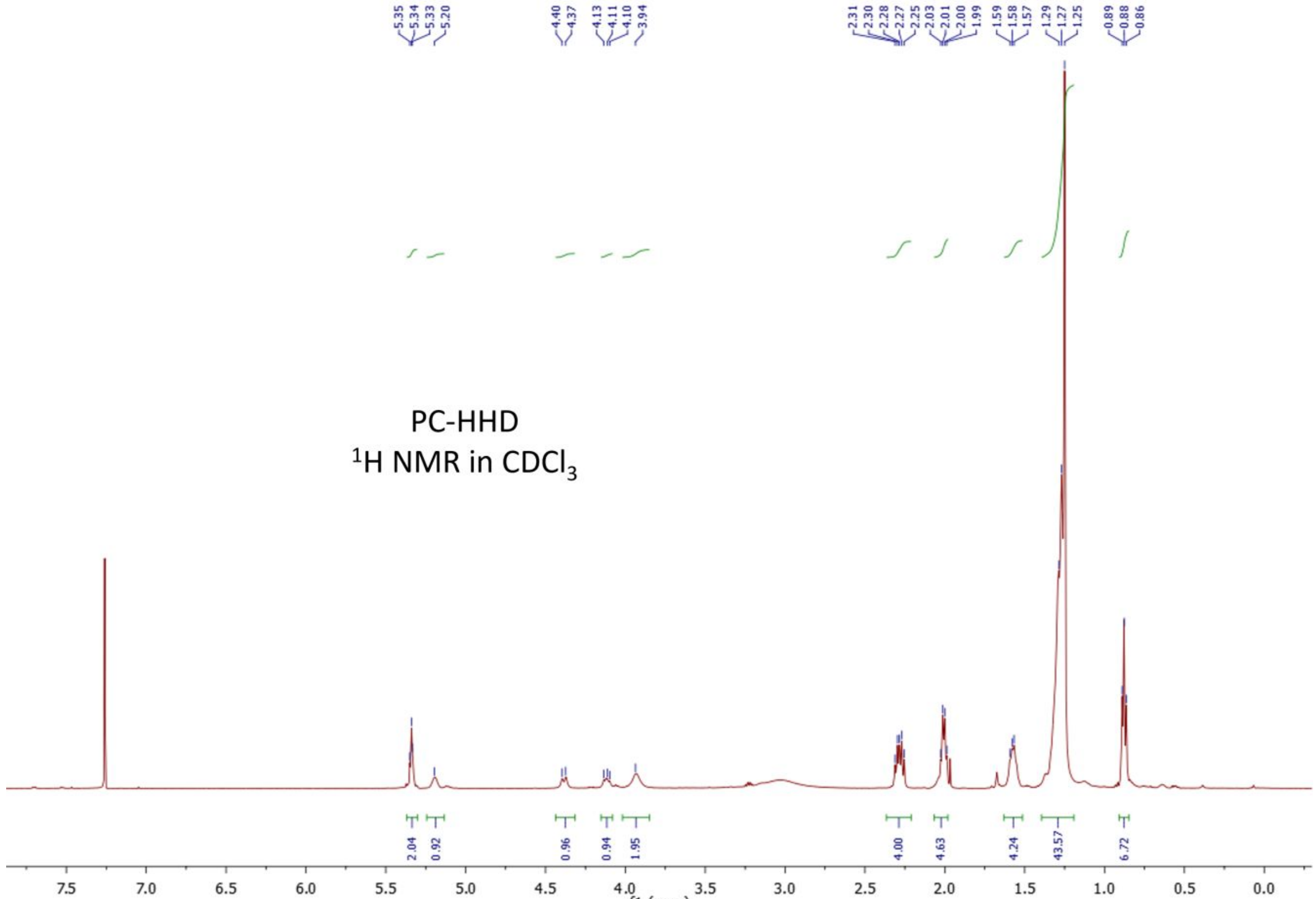

Figure S6. 1H NMR spectrum of PC-HHD in $CDCl_3$

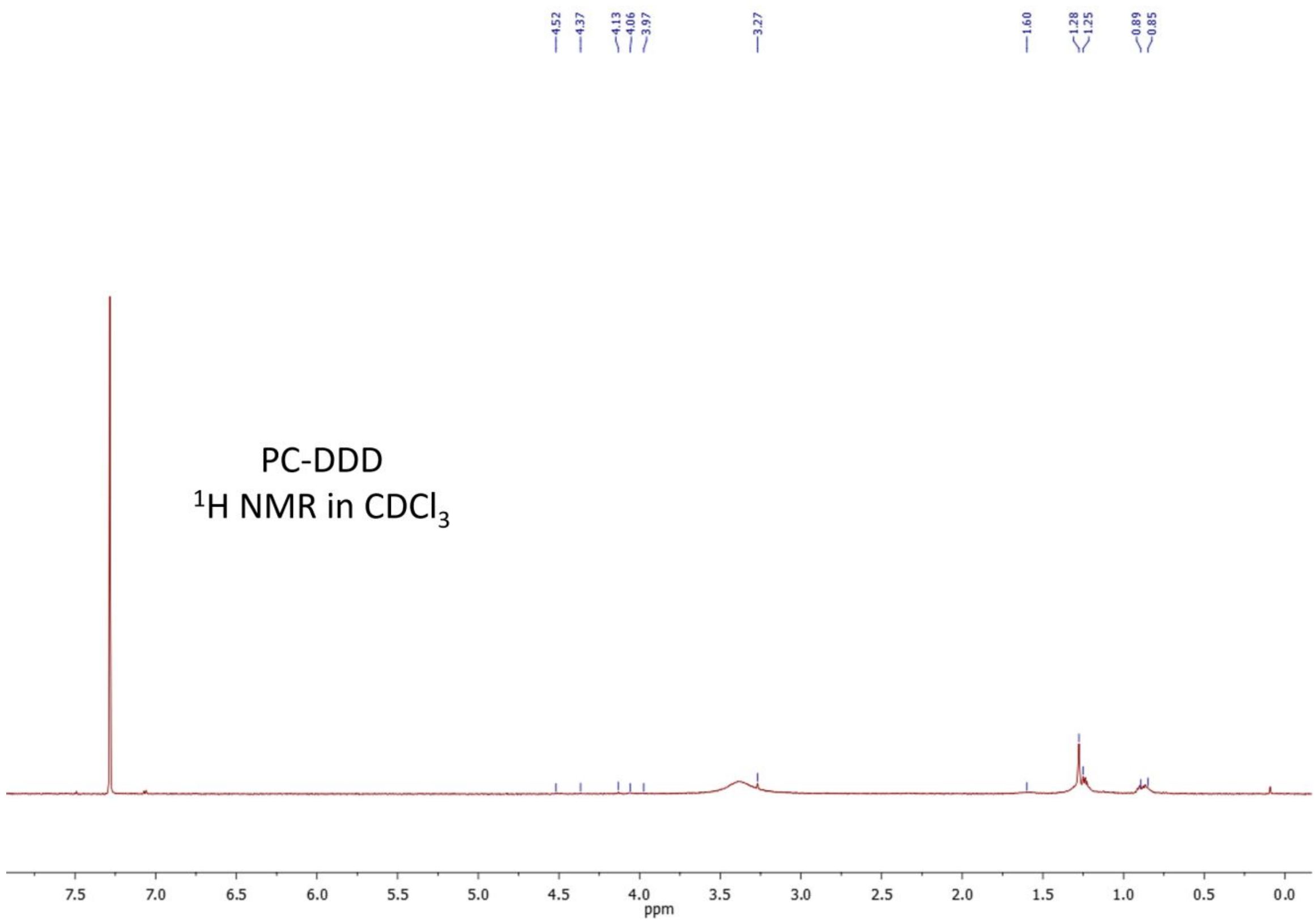

Figure S7. 1H NMR spectrum of PC-DDD in $CDCl_3$

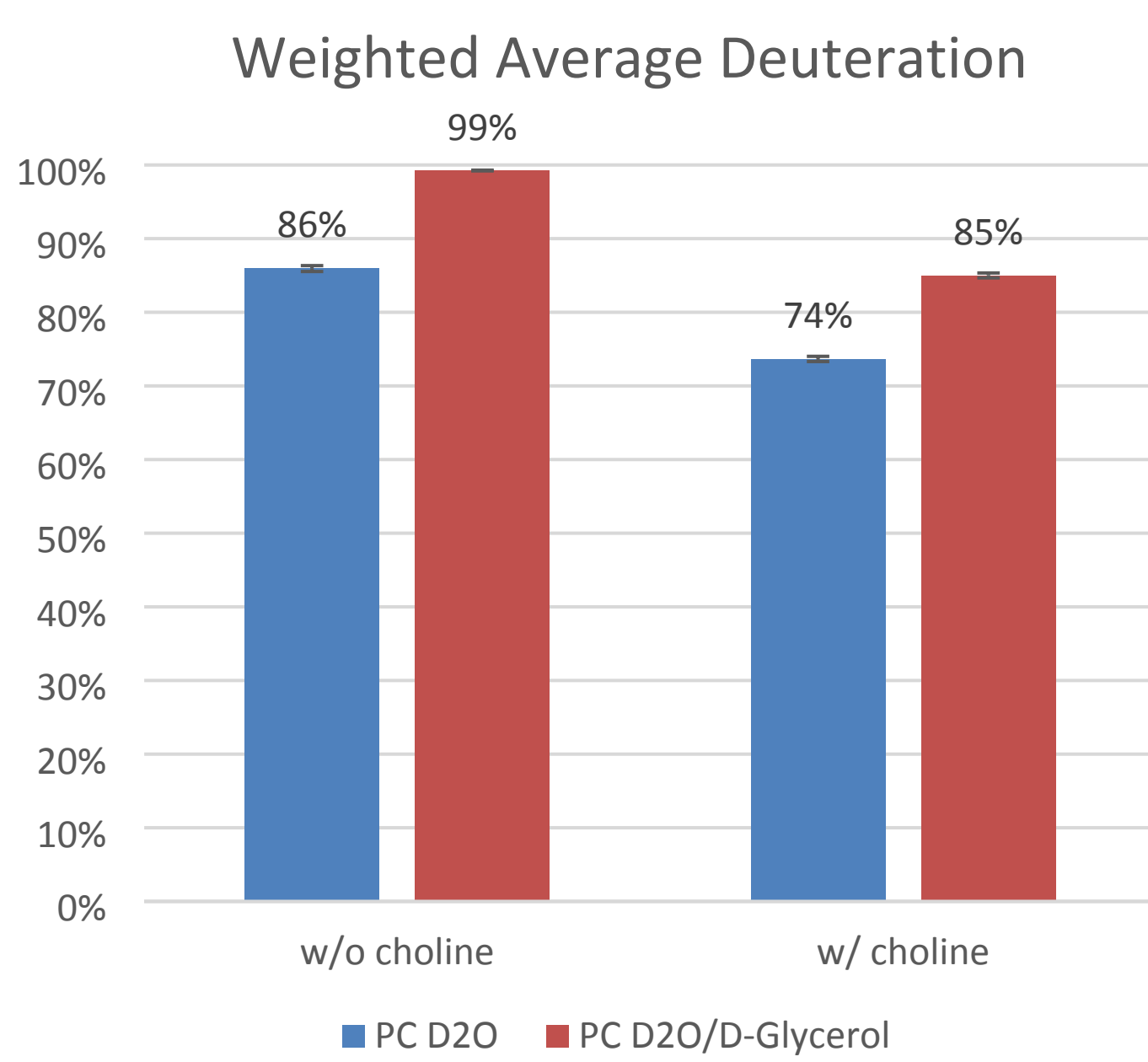

Figure S8. Average deuteration level of PC-DHH (Blue) and PC-DDH (red) calculated from MS analysis. The average deuteration level from the left two columns (86% and 99%) was calculated based on PC without choline part. The average deuteration level from the right two columns (74% and 85%) was calculated based on the whole PC molecules.

Table S1. Average deuteration level of each PC components in PC-DHH and PC-DDH calculated by MS analysis. Similar deuteration level was observed for each PC molecule, suggesting no discrimination of deuteration on PC structure.

Lipid	D2O w/o choline	D2O/D-glycerol w/o choline	D2O w/ choline	D2O/D-glycerol w/ choline
**PC 24:0**	85.4%	97.9%	71.0%	81.4%
**PC 30:0**	86.8%	99.3%	74.1%	84.8%
**PC 28:0**	86.8%	99.3%	73.6%	84.1%
**PC 32:0**	86.6%	99.2%	74.5%	85.3%
**PC 32:1**	85.6%	99.2%	73.4%	85.0%
**PC 37:2**	85.6%	99.3%	74.3%	86.2%
**PC 38:2**	85.4%	99.3%	74.4%	86.5%
**PC 36:1**	86.3%	99.2%	75.0%	86.2%
**PC 36:2**	85.6%	99.2%	74.1%	85.9%
**PC 35:2**	84.9%	99.3%	73.2%	85.7%
**PC 34:2**	85.1%	99.2%	73.2%	85.3%
**PC 35:1**	85.8%	99.9%	74.3%	86.5%
**PC 34:1**	85.8%	99.2%	74.1%	85.6%
**PC 34:0**	87.0%	99.2%	75.3%	85.9%
**PC 33:2**	84.7%	99.2%	72.6%	85.1%
**PC 33:1**	85.1%	99.3%	73.2%	85.4%
**PC 32:2**	85.7%	99.2%	73.2%	84.7%
**PC 31:1**	86.7%	99.2%	74.0%	84.7%
**PC 30:1**	86.1%	99.2%	73.2%	84.4%
**PC 30:2**	85.6%	99.2%	72.5%	84.0%
**PC 26:0**	86.2%	99.2%	72.4%	83.3%

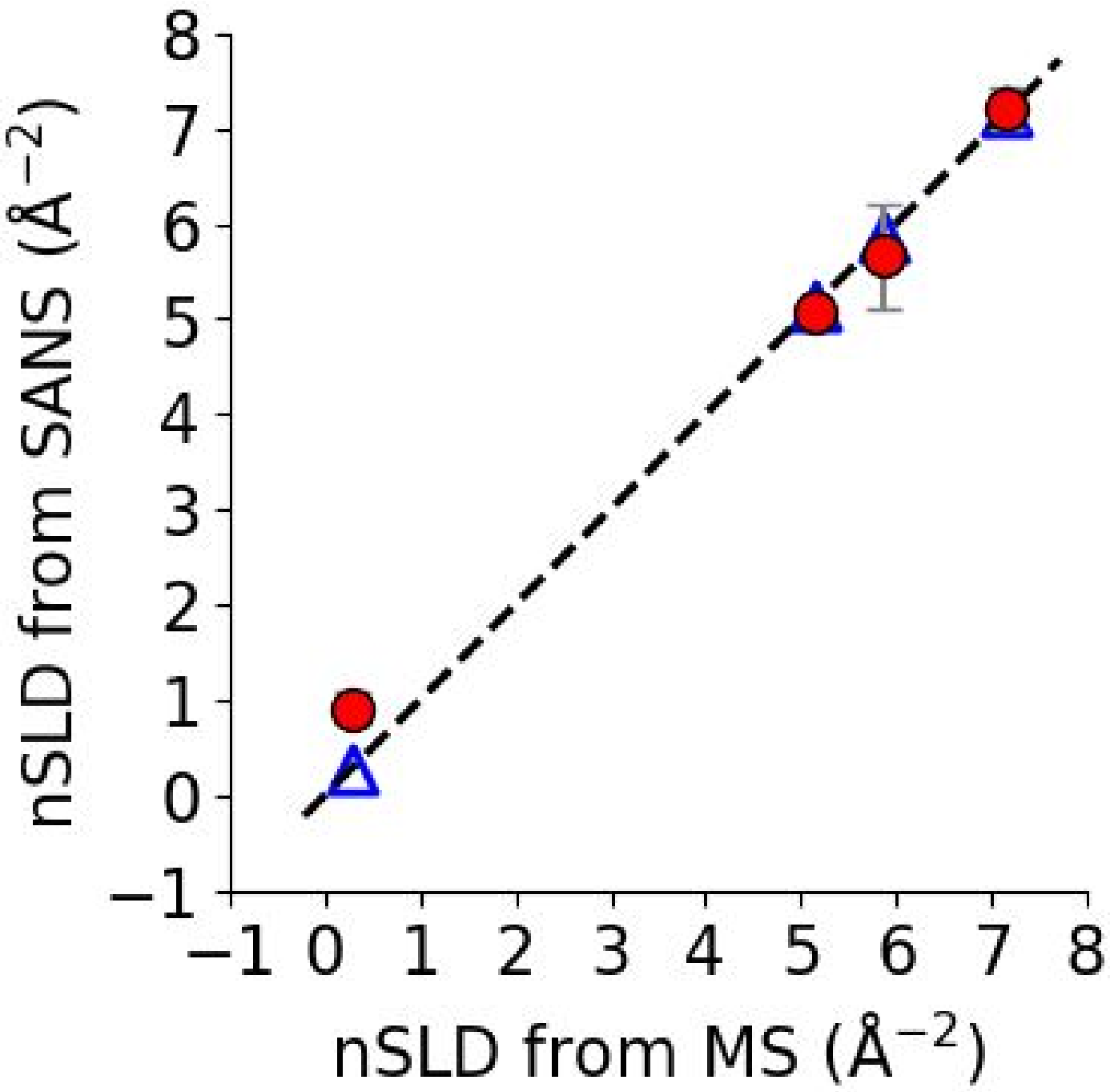

Figure S9. Comparison of neutron scattering length densities for H- and D-PC lipids measured by MS (red filled circles) and SANS (open triangles). The dashed black line represents the ideal 1:1 correlation.

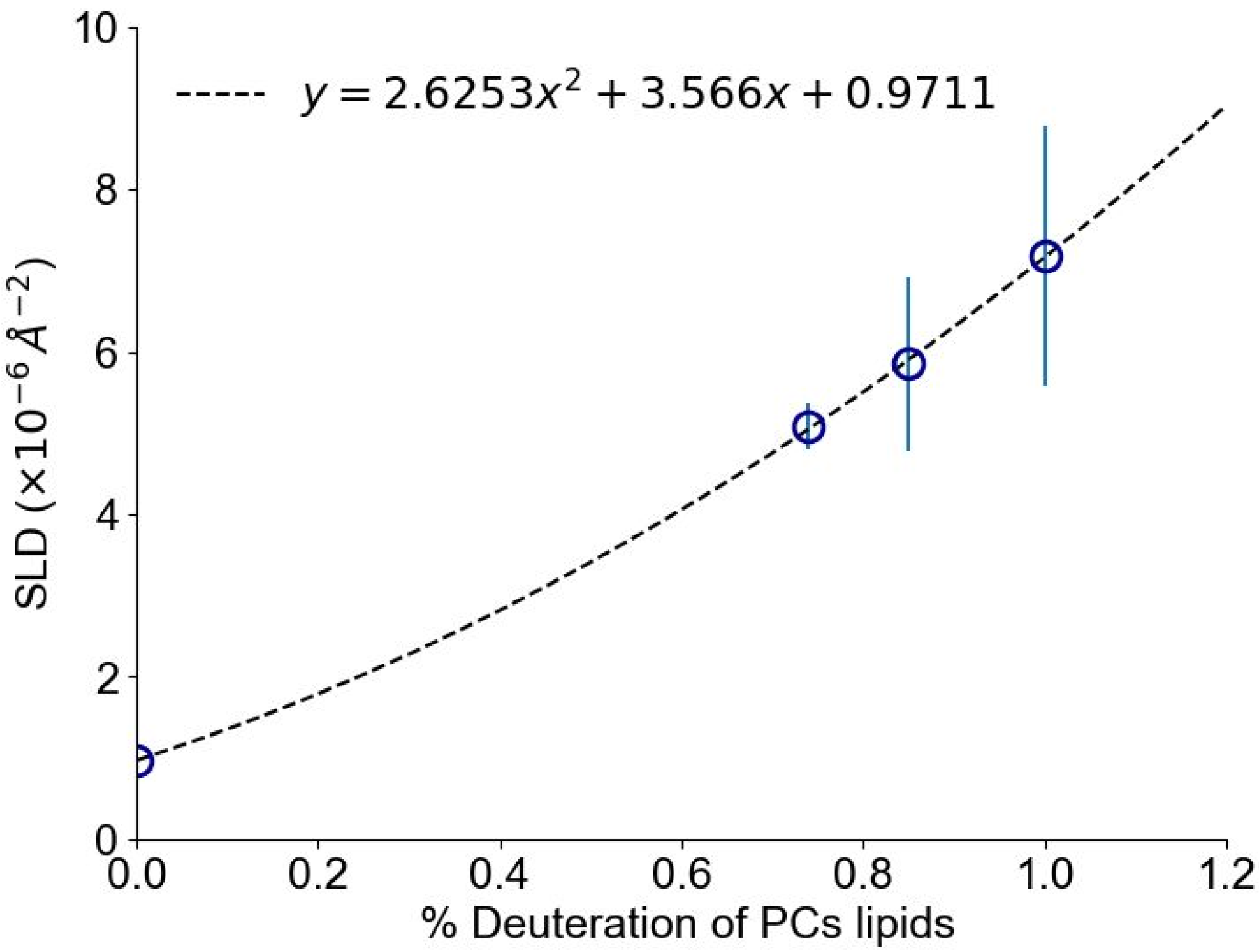

Figure S10. Neutron scattering length density versus level of deuteration obtained from mass spectrometry. The data was fitted with a second order polynomial function.

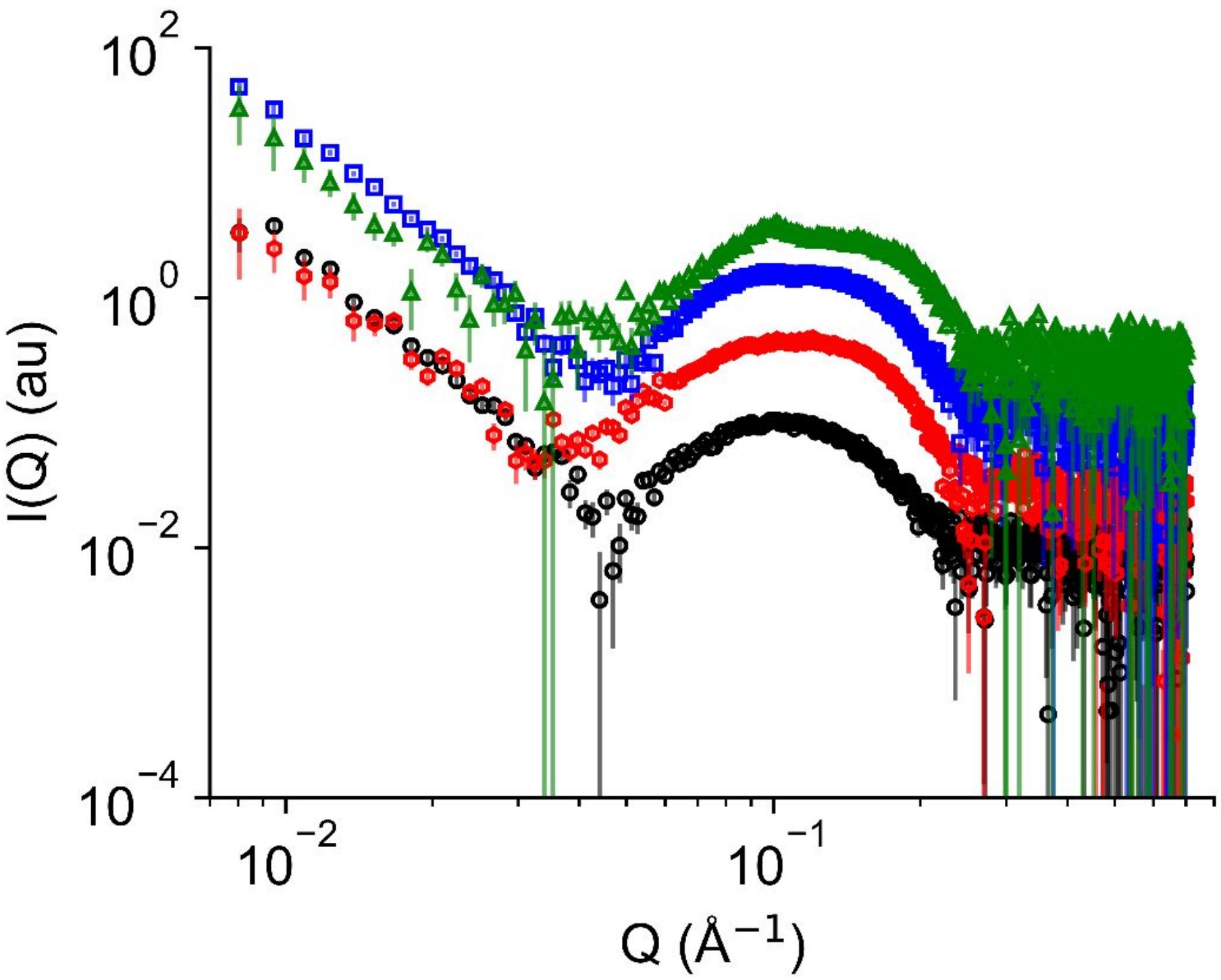

Figure S11. SAXS data for deuterium labeled PC lipids. PC-HHH (black); PC-DHH (red); PC-DDH (blue); and PC-DDD (green).
